# Differential Brain MicroRNA Expression Profiles After Acute and Chronic Infection of Mice With *Toxoplasma gondii* Oocysts

**DOI:** 10.3389/fmicb.2018.02316

**Published:** 2018-10-02

**Authors:** Rui-Si Hu, Jun-Jun He, Hany M. Elsheikha, Fu-Kai Zhang, Yang Zou, Guang-Hui Zhao, Wei Cong, Xing-Quan Zhu

**Affiliations:** ^1^State Key Laboratory of Veterinary Etiological Biology, Key Laboratory of Veterinary Parasitology of Gansu Province, Lanzhou Veterinary Research Institute, Chinese Academy of Agricultural Sciences, Lanzhou, China; ^2^College of Veterinary Medicine, Northwest A&F University, Yangling, China; ^3^Faculty of Medicine and Health Sciences, School of Veterinary Medicine and Science, The University of Nottingham, Loughborough, United Kingdom; ^4^College of Marine Science, Shandong University at Weihai, Weihai, China

**Keywords:** *Toxoplasma gondii*, oocysts, cerebral toxoplasmosis, deep sequencing, microRNAs differential expression

## Abstract

Brain microRNAs (miRNAs) change in abundance in response to *Toxoplasma gondii* infection. However, their precise role in the pathogenesis of cerebral infection with *T. gondii* oocyst remains unclear. We studied the abundance of miRNAs in the brain of mice on days 11 and 33 post-infection (dpi) in order to identify miRNA pattern specific to early (11 dpi) and late (33 dpi) *T. gondii* infection. Mice were challenged with *T. gondii* oocysts (Type II strain) and on 11 and 33 dpi, the expression of miRNAs in mouse brain was investigated using small RNA (sRNA) sequencing. miRNA expression was confirmed by quantitative reverse transcription polymerase chain reaction (qRT-PCR). Gene Ontology (GO) enrichment and Kyoto Encyclopedia of Genes and Genomes (KEGG) pathway analysis were performed to identify the biological processes, molecular functions, and cellular components, as well as pathways involved in infection. More than 1,500 miRNAs (1,352 known and 150 novel miRNAs) were detected in the infected and control mice. The expression of miRNAs varied across time after infection; 3, 38, and 108 differentially expressed miRNAs (*P* < 0.05) were detected during acute infection, chronic infection and chronic vs. acute infection, respectively. GO analysis showed that chronically infected mice had more predicted targets of dysregulated miRNAs than acutely infected mice. KEGG analysis indicated that most predicted targets were involved in immune- or disease-related pathways. Our data indicate that *T. gondii* infection alters the abundance of miRNAs in mouse brain particularly at the chronic stage, probably to fine-tune conditions required for the establishment of a latent brain infection.

## Introduction

The intracellular protozoan parasite *Toxoplasma gondii* is an opportunistic pathogen, which can virtually infect and replicate within any nucleated cells of warm-blooded animals and humans (Jones and Dubey, 2012). *T. gondii* has a complex life cycle that includes asexual propagation and the formation of tachyzoites and bradyzoites-containing cysts in the intermediate host (Black and Boothroyd, 2000) and sexual reproduction and formation of oocysts in the intestinal epithelium of felids (Tenter et al., 2000). Humans can be infected through (i) ingesting undercooked meat containing *T. gondii* tissue cysts, (ii) drinking water contaminated with sporulated oocysts, and (iii) transplacental (vertical) transmission (Elsheikha, 2008). This zoonotic pathogen infects approximately one-third of the world population and can cause a variety of clinical symptoms and even death in immuno-compromised patients (e.g., AIDS patients and organ transplant recipients) and in fetuses of naïve women infected during pregnancy (Dubey, 2008; Elsheikha, 2008; Zhou et al., 2011).

Previous studies have reported a correlation between a deregulated immunoinflammatory response and brain dysfunction in infected humans and animals (Cannella et al., 2014; Blanchard et al., 2015; Elsheikha and Zhu, 2016; Marra, 2018). Effective therapeutic interventions to control brain infection are therefore desirable and will be facilitated by a better understanding of the host immune response against the parasite. However, knowledge about the molecular mechanisms underlying the deregulation of immune responses observed in oocyst-induced cerebral toxoplasmosis remains limited. Oocysts have remarkable ability to endure in the environment (Lindsay and Dubey, 2009; Torrey and Yolken, 2013). Infection acquired through the consumption of water contaminated with *T. gondii* oocysts has been frequently reported (Dubey, 2004; Hill et al., 2011), and can be linked to even waterborne outbreaks (Boyer et al., 2011).

MicroRNA (miRNA) profiling has emerged as a useful approach to study the pathogenesis of many protozoan species, such as *Cryptosporidium, Plasmodium*, and *Toxoplasma* (Judice et al., 2016). Also, miRNAs have been promising biomarkers for the diagnosis and monitoring of progression of parasitic diseases (Hoy et al., 2014; Cai et al., 2015). miRNAs are short (20–24 nucleotides) endogenous non-coding, single stranded, RNA sequences that can control gene expression at the posttranscriptional level and mediate regulatory signals between cells in health and disease (Bartel, 2004; Triboulet et al., 2007; Hobert, 2008; Winter et al., 2009; Zeiner et al., 2010). *T. gondii* infection requires specific miRNA for efficient replication (Zeiner et al., 2010; Cong et al., 2017) and can manipulate host signaling pathways (Hakimi and Ménard, 2010). Infection with the parasite tissue cysts and tachyzoites can alter miRNA expression in the brain (Xu et al., 2013) and spleen (He et al., 2016) of the host, respectively. However, miRNA expression patterns in the mouse brain during acute and chronic stages of infection with *T. gondii* oocysts is unknown.

We previously reported, using next-generation sequencing technology, the differential expression of miRNAs in response to *T. gondii* infection in mouse liver (Cong et al., 2017) and brain (Xu et al., 2013). To our knowledge, no other study has yet explored the possible role of miRNAs in murine cerebral toxoplasmosis caused by oocyst infection. Expanding our earlier observations of differential expression of specific miRNAs between healthy and infected mice, we performed small RNA transcriptome sequencing analysis of the mouse brain in response to infection with *T. gondii* oocysts. Our data provide a platform for the design of functional studies to map the function of the differentially expressed miRNA identified in the brain of mice infected with *T. gondii* oocysts. Our findings indicate that specific miRNAs regulate the expression of inflammatory cytokines in the mouse brain in response to infection.

## Materials and Methods

### Ethics Approval

All animal experiments were approved by the Animal Administration and Ethics Committee of Lanzhou Veterinary Research Institute, Chinese Academy of Agricultural Sciences. The animals were handled in compliance with the animal ethics requirements of the People’s Republic of China. Every effort was made to minimize animal suffering during the experiment.

### Production and Purification of Oocysts

One, 10-week-old, specific-pathogen-free, kitten was infected orally with 100 freshly prepared parasite cysts obtained from brain homogenate of Kunming mice infected with *T. gondii* PRU strain. This strain was used because the majority of human toxoplasmosis cases have been associated with type II strains (Howe and Sibley, 1995). The cat feces were examined daily for the presence of oocysts. Once detected, *T. gondii* oocysts were isolated from the feces using sucrose flotation and CsCl gradient, as described previously (Staggs et al., 2009). To induce sporulation, oocysts were centrifuged at 360 × *g* and the oocyst’s pellet was suspended in 2% sulfuric acid and aerated on a shaker for 7 days at ambient temperature. Sporulated oocysts were washed twice with 0.85% saline and suspended in 2% sulfuric acid. Finally, the number of oocysts was determined using hemocytometer and adjusted to 100 oocysts/ml in PBS and stored at 4°C.

### Infection of Mice

Female 7-week-old BALB/c mice were purchased from Lanzhou University Laboratory Animal Centre (Lanzhou, Gansu Province, China). All mice were handled according to protocols approved by the Animal Ethics Committee of Lanzhou Veterinary Research Institute, Chinese Academy of Agricultural Sciences. Mice were housed in an Animal Biosafety Level 2 (ABSL-2) containment laboratory, with temperature-controlled room (22 ± 0.5°C) under 12 h light/dark cycles. Mice were fed a commercial rodent pellet diet and had access to water *ad libitum*. Mice were rested for 1 week before being infected. Twelve mice were randomly divided into four groups (three mice/group): mice infected for 11 days, mice infected for 33 days, control mice for 11 days, and control mice for 33 days. *T. gondii* infection was induced in each mouse via oral inoculation of 100 *T. gondii* oocysts in 1 ml of PBS. The uninfected (control) mice were sham-inoculated with 1 ml of PBS only. Body weight of the mice was measured daily following infection, and all mice were monitored daily for the development of clinical signs characteristics of *T. gondii* infection, such as ruffled hair, neurological manifestations and physical activity. At 11 and 33 dpi, mice were anesthetized by intraperitoneal injection with 100 μl xylazine (20 mg/ml) and ketamine (1 mg/ml) in PBS, brain tissues were harvested and quickly washed in PBS. All brain samples were placed separately in sterile tubes, flash frozen in liquid nitrogen, and stored frozen at -80°C, until used.

### Detection of *T. gondii* in the Brain

TIANamp Genomic DNA kit was used to extract DNA from the brain tissues (TianGen^TM^, Beijing, China) and DNA samples were stored frozen at -20°C. The presence of *T. gondii* in mouse brain was investigated using a semi-nested PCR assay targeting *T. gondii B1* gene (Cong et al., 2016). Positive PCR amplicons were genotyped using PCR-restriction fragment length polymorphism analysis (PCR-RFLP) as previously described (Cong et al., 2015). Samples from mouse brain were collected and fixed in 10% buffered formalin (pH 7.2) for a few days before dehydration through a graded series of alcohol to xylol and embedded in paraffin wax. Sections of 5 μm thick from paraffin wax blocks were cut and stained with hematoxylin and eosin (H & E) for histopathological analysis.

### RNA Extraction

Total RNA was extracted from the brain tissue of infected and non-infected mice at 11 and 33 dpi. Brain tissues were homogenized in 1 ml Trizol reagent (Invitrogen, Carlsbad, CA, United States). The concentration of RNA was determined using Nanodrop 2000 spectrophotometer (Thermo Scientific, United States). The extracted RNA samples were subjected to quality control checks in order to ensure the high quality of sRNA library construction. The purity of the RNA preparation was assessed by calculating the ratio at 260 and 280 nm. All RNA preparations had a ratio of absorbance (260/280 nm) > 1.8. The assay also confirmed that the RNA samples were free of genomic DNA contamination. The RNA integrity was assessed using the RNA Nano 6000 Assay Kit of the Agilent Bioanalyzer 2100 system (Agilent Technologies, Santa Clara, CA, United States). Only RNA samples with the RNA integrity numbers (RINs) > 7 were used for miRNA profiling analysis. The extracted RNA samples were stored frozen at -80°C, until analysis.

### Small RNA Library Preparation and Sequencing

About 3 μg of total RNA per sample was used as input material to construct small RNA (sRNA) sequencing library according to the instructions of NEBNext^®^ Multiplex sRNA Library Prep Set for Illumina^®^ (NEB, United States). Index codes were added to link sequences to the respective sample. Agilent Bioanalyzer 2100 system (Agilent, Santa Clara, CA, USA) was used to assess the quality of the libraries using DNA High Sensitivity Chips. Following the library generation, the coded samples was clustered on a cBot Cluster Generation System using TruSeq SR Cluster Kit v3-cBot-HS (Illumina). After cluster generation, the libraries were sequenced on an Illumina HiSeq X Ten platform and 50 bp single-end reads were generated.

### Bioinformatics Analysis

Custom Perl and Python scripts were used to process the raw reads, where ploy-N, with 5′ adapter contaminants, without 3′ adapter, or the insert tag, containing ploy A or T or G or C, and low-quality reads were removed in order to obtain clean reads. The Q20, Q30, GC content, and the error rate of the clean reads were determined. A length range of 18∼35 nt from clean reads (about 92% of total reads) was chosen to perform all subsequent analyses. The small RNA tags were mapped to the published reference *Mus musculus* genome sequence by Bowtie (Langmead et al., 2009), without mismatch to analyze the expression and distribution of sRNAs. Mapped sRNA tags were used to search for known miRNA. miRbase 20.0 was used as reference, and software mirdeep 2 (Friedländer et al., 2012) and srna-tools-cli were used to identify the potential miRNA.

RepeatMasker and Rfam database were used for sRNA mapping in order to remove non-coding RNAs (tRNA, rRNA, snRNA, and snoRNA), protein-coding genes and repeat gene sequences. The characteristics of hairpin structure of miRNA precursors, miREvo (Wen et al., 2012) and mirdeep2 (Friedländer et al., 2012) were integrated to predict novel miRNA. miRNA counts and base bias on the first position of the identified miRNA with certain length and on each position of all identified miRNAs were identified by custom scripts. After sRNA annotation, miFam.dat^1^ was utilized to search for known miRNA’s families, and novel miRNA precursor was submitted to Rfam^2^ to look for Rfam families, and then explore the occurrence of miRNA families identified from the samples in other families. The prediction of the target genes of miRNAs was performed using miRanda (Betel et al., 2008) and PITA^3^. The quantification of miRNA was evaluated by TPM (Zhou et al., 2010). The DESeq R package (1.8.3) was used for the differential expression analysis (Anders and Huber, 2010). FDR adjusted *P*-value (Benjamini-Hochberg method for multiple corrections) of 0.05 was considered significant.

### GO and KEGG Enrichment Analyses

Gene ontology enrichment analysis of the predicted target genes of the differentially expressed miRNAs (*P* < 0.05) was performed using the GOseq R package, based on a Wallenius non-central hyper-geometric distribution (Young et al., 2010). KEGG^4^ pathway analysis and functional annotation of the predicted target genes were conducted using KOBAS 3.0 software (Mao et al., 2005; Kanehisa et al., 2008).

### Verification of miRNA Expression by qRT-PCR

The data were validated by quantitative reverse transcription PCR (qRT-PCR) analysis of seven of the differentially expressed miRNAs (*P* < 0.05) in the brain sample to confirm gene expression ratios obtained by sequencing. Total RNA from infected and uninfected mouse groups was extracted using Trizol method (Invitrogen, United States) and the quality of RNA template was assessed using a NanoDrop 2000 spectrophotometer (Thermo Scientific, United States). RT-PCR reactions were carried out in biological triplicate for each RNA sample. The extracted RNA was treated with DNase I to remove any residual genomic DNA and then reverse-transcripted into single strand cDNA using Mir-X^TM^ miRNA First-Strand Synthesis Kit (Clontech, Mountain View, CA, United States). SYBR^®^ Premix Ex Taq^TM^ II (Takara, Shiga-ken, Japan) was used to perform qRT-PCR reaction on QIAGEN’s real-time PCR cycler (QIAGEN, Hilden, Germany). The 25 μl qRT-PCR reaction contained 9 μl ddH_2_O, 12.5 μl SYBR Advantage Premix (2X), 0.5 μl ROX Dye (50 X), 0.5 μl of each miRNA-specific forward primer (**Table 1**), 0.5 μl mRQ 3′ Primer, and 2 μl cDNA. The qRT-PCRs were performed under the following cycling conditions: initial denaturation at 95°C for 30 s, followed by 40 cycles of 95°C for 5 s, 60°C for 30 s; melt curve analysis was performed from 60 to 95°C to confirm primer specificity by the presence of single melting curve peak, indicating a single amplicon in each qPCR reaction. No-template control and no-reverse transcriptase control were included in each plate to verify the absence of contamination. Expression of miRNAs was normalized to the level of *U6* small nuclear RNA (snRNA). qRT-PCR analysis was performed using the delta-delta C_t_ method (ΔΔC_t_) to measure the relative abundances of miRNAs (Peltier and Latham, 2008). The data were expressed as mean ± standard deviation (SD). Statistical significance was determined by Student’s *t*-test, with *P*-values of < 0.05 deemed to be statistically significant.

**Table 1:** Oligonucleotides used as primers for miRNA-specific qRT-PCR analysis.

miRNAs	miRNA sequence (5′-3′)^∗^
mmu-miR-155-5p F	CCGCGTTAATGCTAATTGTGATAGGGGT
mmu-miR-204-5p F	CGTTCCCTTTGTCATCCTATGCCT
mmu-miR-146a-5p F	CGCTGAGAACTGAATTCCATGGGTT
mmu-miR-142a-5p F	GCGCGCATAAAGTAGAAAGCACTACT
mmu-miR-7043-3P F	GCGACTGTGCCTCTCTGTTTTCAG
mmu-miR-144-3p F	CCGCGCGTACAGTATAGATGATGTACT
mmu-miR-5114 F	ACTGGAGACGGAAGCTGCAAG
U6 F	GGAACGATACAGAGAAGATTAGC
U6 R	TGGAACGCTTCACGAATTTGCG

^∗^*mRQ 3′ primer was provided by the Mir-X^*TM*^ miRNA First-Strand Synthesis and SYBR^®^ qRT-PCR kit (Clontech) and was used as reverse primer in all Mir-X cDNA qPCRs. F represents forward primer and R represents reverse primer*.

## Results

### Oocysts Infection in the Mouse Brain

*Toxoplasma gondii* infection was confirmed in the brain of infected mice at 11 and 33 dpi by positive PCR results. PCR-RFLP analysis of the positive amplicons of *T. gondii B1* gene revealed a restriction fragment pattern consistent with that of *T. gondii* genotype II. The mouse brain of control group and negative PCR control samples revealed negative results. Histopathological analysis revealed the presence of *T. gondii* cyst in the brain of infected mice at 33 dpi (**Supplementary Figure S1**), whereas the parasite cysts were not detected in the brain of uninfected mice or the brain of mice 11 days post infection.

### miRNA Expression Patterns Associated With Infection

miRNA libraries were successfully prepared from the brain of the infected and uninfected (control) mice. Key characteristics of the obtained sequencing data are summarized (**Table 2**). After selecting the appropriate length of sRNA, the base number distribution interval of clean reads in each sample was between 18∼35 nt; one of the highest proportions was 22 nt length of sRNA. 91.9–93.7% of the reads aligned to the reference *M. musculus* genome. During early and late infection, an unequal number of known and novel miRNAs in infected and uninfected mice was detected (**Supplementary Tables S1**, **S2**).

**Table 2:** Characteristics of the sRNA sequences obtained in the present study.

Mouse groups	Sample code	Raw reads	Clean reads	Bases	Error rate (%)	Q20 (%)^a^	Q30 (%)^b^	GC content (%)
11 days post infection
Infected	I	13,723,453	13,562,928	0.686G	0.01	0.9686	0.9291	48.84
Infected	II	18,544,336	18,245,384	0.927G	0.01	0.977	0.9508	48.68
Infected	III	14,433,684	14,190,848	0.722G	0.01	0.9689	0.9288	48.44
Control	IV	10,419,259	10,237,888	0.521G	0.01	0.9729	0.942	48.61
Control	V	16,971,162	16,541,211	0.849G	0.01	0.9575	0.921	48.62
Control	VI	12,876,002	12,595,020	0.644G	0.01	0.9657	0.9323	49.96
33 days post infection
Infected	VII	10,271,598	10,143,170	0.541G	0.01	0.9648	0.9193	48.7
Infected	VIII	13,822,880	13,609,247	0.691G	0.01	0.969	0.9304	48.44
Infected	IX	13,012,055	12,786,416	0.651G	0.01	0.9631	0.9199	48.48
Control	X	17,410,567	16,970,788	0.871G	0.01	0.9553	0.9169	48.68
Control	XI	13,847,968	13,569,857	0.692G	0.01	0.9728	0.9432	48.89
Control	XII	12,009,258	11,825,447	0.600G	0.01	0.9695	0.9309	49.19

^a^*The percentage of bases with a Phred value > 20*.

^b^*The percentage of bases with a Phred value > 30*.

### Differentially Expressed miRNA During Acute and Chronic Infection

**FIGURE 1:**
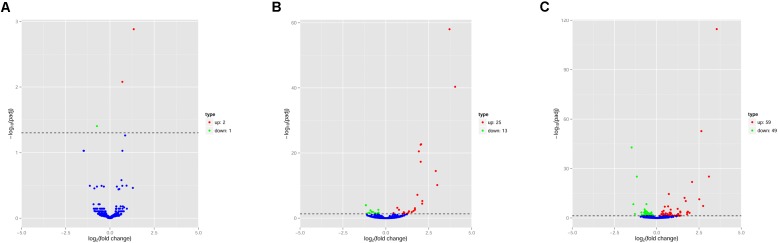
Volcano plots of miRNA expression changes in the mouse brain. Differentially expressed miRNAs (*P* < 0.05) in **(A)** “acute *vs* uninfected,” **(B)** “chronic vs. uninfected” and **(C)** “chronic vs. acute.” Red, green and blue dots represent upregulated, downregulated and non-regulated miRNAs, respectively. The *x*-axis represents the log2 ratio of gene expression levels between mouse groups. The *y*-axis is adjusted *p*- based on -log10.

The pattern of global expression of miRNAs (*P* < 0.05) in the brain of healthy mice was compared with that of acutely and chronically infected mice. Total differentially expressed miRNAs (*P* < 0.05) during acute and chronic infection are summarized (**Supplementary Table S3**). More miRNAs were differentially expressed during chronic compared to acute stage of *T. gondii* infection (**Figure 1**). By comparing acutely infected mice with uninfected mice, 2 miRNAs were upregulated and one miRNA was downregulated (**Table 3**). When comparing chronically infected mice with uninfected mouse, more differentially expressed miRNAs (*P* < 0.05) were detected, including 25 upregulated miRNAs and 13 downregulated miRNAs (**Supplementary Table S3**).

**Table 3:** The top differentially expressed miRNAs (*P* < 0.05) in mouse brains during acute and chronic infection with *Toxoplasma gondii* oocysts.

Mouse groups	miRNA	Fold change	*p*-value	*p*-adjustment	Expression
Acute vs. uninfected					
	mmu-miR-155-5p	1.3422	2.01E-06	0.001312	Up-regulated
	mmu-miR-1983	0.70165	2.55E-05	0.0083223	Up-regulated
	mmu-miR-204-5p	-0.73525	0.0001809	0.039436	Down-regulated
					
Chronic *vs* uninfected					
	mmu-miR-146a-5p	3.7084	1.21E-61	9.77E-59	Up-regulated
	mmu-miR-155-5p	4.0375	1.12E-43	4.54E-41	Up-regulated
	mmu-miR-142a-3p	2.0541	1.00E-25	2.03E-23	Up-regulated
	mmu-miR-142b	2.0541	1.00E-25	2.03E-23	Up-regulated
	mmu-miR-203-3p	2.0275	1.77E-25	2.87E-23	Up-regulated
	mmu-miR-21a-5p	1.9238	2.31E-23	3.11E-21	Up-regulated
	mmu-miR-142a-5p	2.0311	4.23E-20	4.88E-18	Up-regulated
	mmu-miR-147-3p	2.9077	3.22E-17	3.25E-15	Up-regulated
	mmu-miR-5107-3p	2.9955	7.65E-13	6.86E-11	Up-regulated
	mmu-miR-223-3p	1.838	8.64E-10	6.98E-08	Up-regulated
	mmu-miR-219b-3p	-1.1693	1.63E-06	0.0001015	Down-regulated
	mmu-miR-219a-5p	-1.1559	1.97E-06	0.0001137	Down-regulated
	mmu-miR-32-5p	-0.43524	5.90E-05	0.0026573	Down-regulated
	mmu-miR-33-5p	-0.89719	9.15E-05	0.0036973	Down-regulated
	mmu-miR-99a-3p	-0.83122	0.00034778	0.012218	Down-regulated
	mmu-miR-199b-5p	-0.74691	0.00037218	0.01253	Down-regulated
	mmu-miR-326-3p	-0.42889	0.00071845	0.020732	Down-regulated
	mmu-miR-3081-3p	-0.97471	0.00078346	0.021829	Down-regulated
	mmu-miR-144-3p	-0.63175	0.0010824	0.027398	Down-regulated
	mmu-miR-136-5p	-0.50052	0.0015125	0.035944	Down-regulated
Chronic vs. acute					
	mmu-miR-146a-5p	3.5491	3.24E-118	2.38E-115	Up-regulated
	mmu-miR-142a-3p	2.6381	7.90E-56	1.93E-53	Up-regulated
	mmu-miR-142b	2.6381	7.90E-56	1.93E-53	Up-regulated
	mmu-miR-155-5p	3.0876	5.94E-28	7.26E-26	Up-regulated
	mmu-miR-142a-5p	2.0908	1.26E-24	1.32E-22	Up-regulated
	mmu-miR-423-5p	0.71476	2.92E-17	2.68E-15	Up-regulated
	mmu-miR-21a-5p	1.6451	7.42E-15	6.04E-13	Up-regulated
	mmu-miR-147-3p	2.5151	5.69E-14	4.17E-12	Up-regulated
	mmu-miR-10a-5p	1.7159	7.83E-13	5.21E-11	Up-regulated
	mmu-miR-5107-3p	2.7461	9.31E-10	4.87E-08	Up-regulated
	mmu-miR-412-5p	-1.4876	8.50E-46	1.56E-43	Down-regulated
	mmu-miR-1983	-1.1776	5.92E-28	7.26E-26	Down-regulated
	mmu-miR-379-5p	-0.61196	6.21E-11	3.79E-09	Down-regulated
	mmu-miR-1197-3p	-1.3826	7.61E-11	4.29E-09	Down-regulated
	mmu-miR-322-3p	-0.71614	1.41E-07	5.74E-06	Down-regulated
	mmu-miR-409-5p	-0.63403	1.95E-06	6.49E-05	Down-regulated
	mmu-miR-674-3p	-0.6952	1.87E-06	6.49E-05	Down-regulated
	mmu-miR-132-5p	-0.58255	5.81E-06	0.0001639	Down-regulated
	mmu-miR-127-3p	-0.31406	1.48E-05	0.0003868	Down-regulated
	mmu-miR-3081-3p	-0.90774	1.73E-05	0.000422	Down-regulated

**FIGURE 2:**
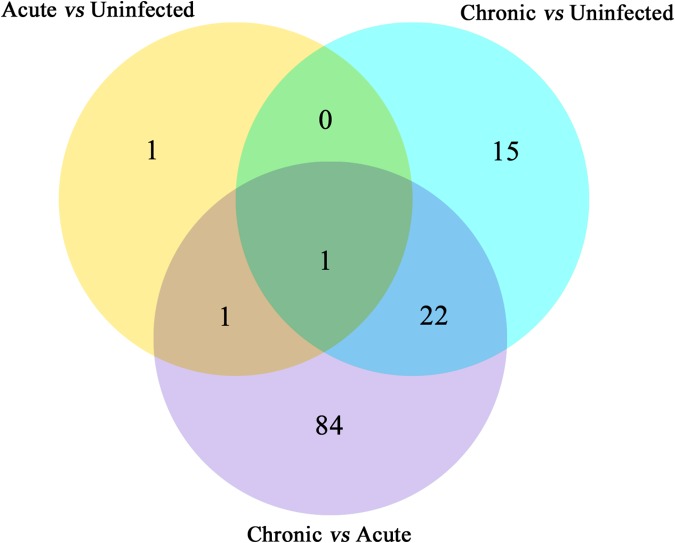
Venn diagram showing the number of commonly expressed and specifically expressed miRNAs between mouse groups. The miRNAs that are significant and specific for in mice with acute infection vs. healthy control are shown in the yellow circle. The light blue circle represents the miRNA markers that discriminate chronically infected mice and control mice, while the purple circle stands for the miRNA markers that permit a distinction of chronically vs. acutely infected mice.

Next, we compared the abundance of miRNA during acute and chronic infection in order to determine the temporal changes in the expression of miRNAs during the course of infection. Out of the 108 differentially expressed miRNAs (*P* < 0.05), 59 were up-regulated and 49 were down-regulated. The differentially expressed miRNAs (*P* < 0.05) in early and late *T. gondii* infection are shown (**Figure 2**). The number of differentially expressed miRNAs (*P* < 0.05) increased from 3 at 11 dpi to 38 at 33 dpi as shown in **Supplementary Figure S2**. These data indicate that more altered expression of miRNAs characterize the brain response to chronic *T. gondii* infection.

**FIGURE 3:**
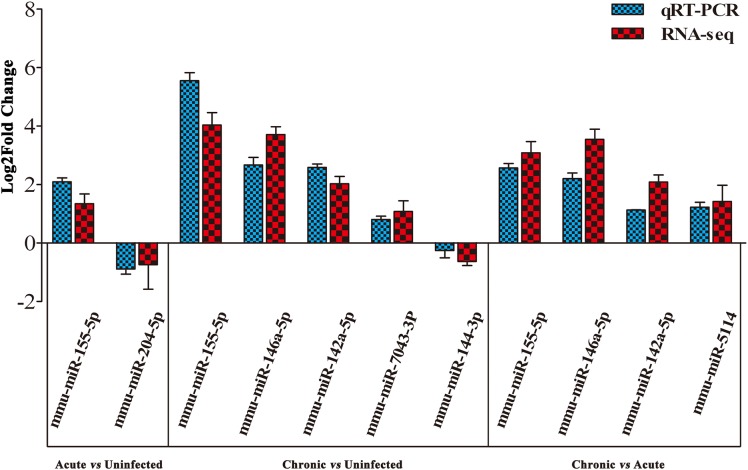
Validation of the differentially expressed miRNAs using qRT-PCR. A bar graph showing the agreement in the expression levels of a panel of seven miRNAs between qRT-PCR and the sequencing data. Columns and error bars indicate means and standard deviations of relative expression levels (*n* = 3), respectively. *X*-axis, represents the differentially expressed miRNAs (*P* < 0.05). The y-axis shows the relative expression levels (log_2_ fold change).

To validate the deep sequencing results, qRT-PCR was carried out on seven selected miRNAs showing different levels of expression (i.e., 5 upregulated and 2 downregulated) in the brain of mice. These included: mmu-miR-155-5p, mmu-miR-204-5p, mmu-miR-146a-5p, mmu-miR-142a-5p, mmu-miR-7043-3P, mmu-miR-144-3p, and mmu-miR-5114. As shown in **Figure 3**, qRT-PCR results of the seven examined miRNAs were consistent with those obtained by sequencing, confirming the correctness of the obtained miRNA data.

### Functional Annotation and KEGG Pathway Enrichment Analysis of miRNA Target Genes

To better understand the roles of the identified miRNAs in mouse brain response to infection, the target genes of miRNAs were identified using the computational prediction tools miRanda and PITA. We performed GO and KEGG enrichment analyses to identify the biological functions of the differentially expressed miRNAs (*P* < 0.05) during acute and chronic infection. The results revealed a total of 33 target genes of the three miRNAs of “acute vs. uninfected,” 719 target genes of the 38 miRNAs of “chronic vs. uninfected,” and 1,911 target genes of the 108 miRNAs of “chronic vs. acute.” We found that, in “acute vs. uninfected” mice, two upregulated miRNAs were successfully assigned to 14 significantly enriched GO terms, and one downregulated miRNA was significantly enriched in 21 GO terms (corrected *P* ≤ 0.05). Among these significantly enriched GO terms, we found that the upregulated miRNAs were significantly enriched in negative regulation of calcium ion transmembrane transporter activity, negative regulation of calcium ion transmembrane transport, negative regulation of ion transmembrane transporter activity, negative regulation of calcium ion transport, and one downregulated gene was significantly enriched in the cell part, cell, biological regulation, and intracellular membrane-bounded organelle.

Regarding “chronic infection vs. uninfected” mice, we detected 25 upregulated miRNAs that were successfully assigned to 652 significantly enriched GO terms, and 13 downregulated miRNAs that were significantly enriched in 532 GO terms (corrected *P* ≤ 0.05). Among these significantly enriched GO terms, many upregulated miRNAs were enriched in cell part, cell, intracellular part, intracellular, and many downregulated genes were enriched in the cell, cell part, intracellular part, and intracellular.

In “chronic vs. acute” infection, 59 upregulated miRNAs were assigned to 879 significantly enriched GO terms, and 49 downregulated miRNAs were enriched in 1,172 GO terms (corrected *P* ≤ 0.05). Among these significantly enriched GO terms, many upregulated miRNAs were enriched in cell part, cell, intracellular and intracellular part, and many downregulated genes were enriched in the cell part, cell, single-organism process, and intracellular.

**FIGURE 4:**
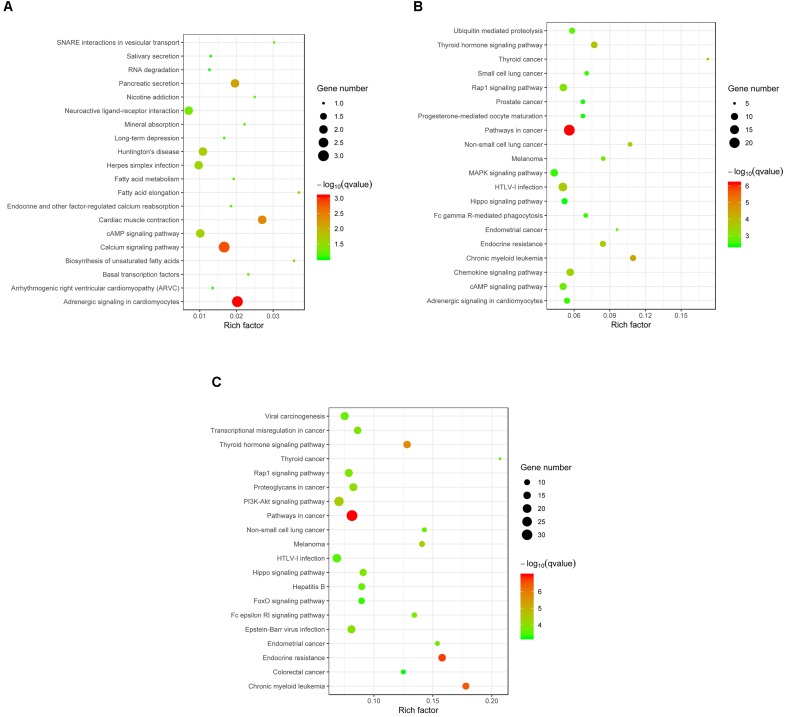
KEGG analysis of the differentially expressed miRNAs (*P* < 0.05) revealed significant enrichment in immune-related pathways. The top 20 enriched pathways of the differentially expressed miRNAs are presented for **(A)** “Acute vs. Uninfected,” **(B)** “Chronic vs. Uninfected” and **(C)** “Chronic vs. Acute.” The degree of KEGG enrichment was determined by the enrichment factor, *q*-value, and miRNA gene target number. The sizes and colors of the spots represent the number of predicted gene targets of the differentially expressed miRNAs (*P* < 0.05) and the *q*-value, respectively.

Target genes of the differentially expressed miRNA were mapped to terms in the KEGG database to identify signaling pathways operating during acute and chronic infection. A total of 33 target genes were enriched in 52 KEGG pathways in “acute vs. uninfected” mice, 719 were enriched in 230 KEGG pathways in “chronic vs. uninfected,” and 911 were enriched in 255 KEGG pathways in “chronic vs. acute”. The top 20 highly enriched pathways in acute infection are shown in **Figure 4A**. such as adrenergic signaling in cardiomyocytes, calcium signaling pathway, and cardiac muscle contraction. The top 20 highly enriched pathways during chronic infection (**Figure 4B**), included immune-related pathways, such as chemokine signaling pathway, Rap1 signaling pathway, cAMP signaling pathway, MAPK signaling pathway, Fc gamma R-mediated phagocytosis, and Hippo signaling pathway. Other pathways associated with disease, such as pathways in cancer, chronic myeloid leukemia, HTLV-I infection were also detected. The top highly enriched pathways in “chronic vs. acute” were also involved in pathways, such as cancer, endocrine resistance and chronic myeloid leukemia (**Figure 4C**). These results suggest that infection with *T. gondii* oocysts stimulates the expression of gene targets at miRNA levels in the mouse brain especially during chronic infection and that miRNAs play key roles in mouse brain immune response to *T. gondii* infection.

## Discussion

Successful outcome of *T. gondii* infection depends on the parasite ability to subvert host immune response, and to survive and replicate in a hostile host environment. However, the mechanisms by which this parasite interferes with host immunity in the brain remain poorly understood. MicroRNAs (miRNAs) have been established as key regulators of various biological processes with possible involvement in the pathobiology of *T. gondii* infection (Hakimi and Ménard, 2010). *T. gondii* infection is capable of modulating host microRNAs (miRNAs) in order to establish conditions that work in favor of parasite growth (Xu et al., 2013; Cong et al., 2017). In agreement with previous work, deep-sequencing analysis of miRNAs of mouse brain at 11 and 33 dpi revealed significant changes in the abundance of miRNAs attributed to infection.

Our analysis revealed > 1,500 miRNAs in both infected and uninfected mouse groups, and some of these were differentially expressed in the infected brains compared with control brain samples. For example, three differentially expressed miRNAs (*P* < 0.05) were detected during acute infection, whereas 38 differentially expressed miRNAs (*P* < 0.05) were detected in chronic infection, indicating that miRNA expression in mouse brain was influenced by the length of time elapsed after infection (Pittman and Knoll, 2015). There were 108 differentially expressed miRNAs (*P* < 0.05) between acutely and chronically infected mice. Although miRNAs in mouse brain tissue challenged with *T. gondii* type II cyst (PRU strain) at duration of infection of 14 and 21 dpi have been previously reported (Xu et al., 2013), the present study demonstrated that the infecting stage and the duration of infection can influence the expression of miRNAs in mouse brain (**Supplementary Table S4**). Some of the upregulated miRNAs (mmu-miR-155-5p, mmu-miR-146a-5p, mmu-miR-142a-3p, mmu-miR-142b, mmu-miR-21a-5p) and the down-regulated miRNAs (mmu-miR-409-5p, mmu-miR-127-3p, mmu-miR-493-5p) (**Table 3**) have been reported in previous studies (He et al., 2016; Cong et al., 2017), indicating that *T. gondii* effect on host miRNAs is not strictly tissue-specific.

The top 10 most differentially abundant miRNAs in chronic infection, as shown in **Table 3**, were the most overexpressed (3–4-fold increase) in chronic infection. The mmu-miR-155-5p was shown to be abundantly expressed in immune cells involved in mouse inflammatory responses, through inhibiting SOCS1 to enhance IFN-α (Rao et al., 2014) and decrease TNF-α (Huck et al., 2017). Also, the upregulation of mmu-miR-146a-5p is relevant to myeloid cell proliferation, inflammation and cancer (Boldin et al., 2011). The expression of the immunomodulatory mmu-miR-155-5p and mmu-miR-146a-5p was shown to be strain-specific and miR-146a ablation can affect early parasite burden, leading to significant differences in IFN-γ production and better survival in C57BL/6 mice (Cannella et al., 2014). Therefore, downregulation of miR-146a in acute infection has probably reduced parasite burden during early infection in order to limit CNS colonization and parasite cyst burden. mmu-miR-142a-3p, mmu-miR-142a-5p, and mmu-miR-142b, were also over-expressed. Of note, mmu-miR-142, plays significant roles in immune regulation (Sun et al., 2011, 2013, 2015), and in the development of nasopharynx carcinoma, cell cycling or IL-6 modulation in hematopoietic cell tissues, mature T cell proliferation and endotoxin-induced mortality in inflammatory processes; However, the roles of the miRNAs described above except mmu-miR-155-5p and mmu-miR-146a-5p in the process of *T. gondii* colonization remain to be elucidated.

KEGG pathway enrichment analysis based on the predicted target genes revealed that dysregulated miRNAs in the brain of infected mice was dominated by an immunological signature, in agreement with the results obtained in *T. gondii* brain infection with tissue cysts (Xu et al., 2013). We found that 255 biological pathways, especially immune-related and disease-related signaling pathways, were abundant among the significantly enriched mRNAs. The dominance of the immune-related signaling pathways indicates that *T. gondii* oocysts establish brain infection by altering host immune response in order to persist in the brain by forming a latent infection (Hunter and Sibley, 2012).

Previous studies suggested that miRNAs do not only regulate host cell development (Taganov et al., 2006; Kim et al., 2008), but were also involved in cancer or infection (O’Connell et al., 2007; Triboulet et al., 2007; Xiao and Rajewsky, 2009). Therefore, we studied the altered pathways of the predicted gene targets of the differentially expressed miRNAs (*P* < 0.05) in acute and chronic infection. Although the dysregulated miRNAs induced by early *T. gondii* infection were enriched in both disease- and signaling-related pathways (**Figure 4A**), the predicted targets of the upregulated or downregulated miRNAs were not significantly enriched (corrected *P* > 0.05). During chronic infection and “chronic vs. acute,” more disease-related pathways were involved in various physiological systems and molecular processes (**Figures 4B,C**). Interestingly, the targets of the upregulated miRNAs were mainly involved in cancer-related pathways (corrected *P* ≤ 0.05), such as pathways in cancer, chronic myeloid leukemia, and thyroid cancer; but the targets of downregulated miRNA were mainly enriched in signaling pathways (corrected *P* ≤ 0.05), such as Fc epsilon RI signaling pathway, cAMP signaling pathway and sphingolipid signaling pathway. These results further substantiate the important roles of miRNA in modulating brain response of mice challenged with *T. gondii* oocysts especially during chronic stage of infection.

## Conclusion

The present study provides a comparative analysis of RNA sequencing-based miRNA differential expression patterns in mouse brain infected with *T. gondii* oocysts during acute and chronic infection. We identified 1,352 known miRNAs and 150 novel miRNAs. Three miRNAs were identified in mouse brain during acute infection and 38 miRNAs during chronic infection. These miRNAs are involved in the regulation of disease-related pathways and immune signaling pathways. Validation of seven differentially regulated miRNAs by qRT-PCR confirmed the validity of the alteration of their expression using RNA-seq analysis. Future studies are needed to further examine the miRNA changes in host brain and to investigate the functional roles of these differently expressed miRNAs, which may contribute to the study of regulatory signaling networks involved in the development of cerebral toxoplasmosis and may provide targets for therapeutic interventions.

## Availability of Data and Materials

The RNA-seq data reported in the present study have been submitted to NCBI SRA database (accession number PRJNA418218). All other data supporting the findings, are available within the paper and its **Supplementary Information Files**.

## Author Contributions

X-QZ and WC conceived and designed the experiments. R-SH, J-JH, F-KZ, YZ, and WC performed the experiments. R-SH, J-JH, HE, and WC contributed reagents, materials and analysis tools. R-SH and WC analyzed the data and wrote the paper. HE, WC, G-HZ, and X-QZ critically revised the manuscript. All the authors read and approved the final version of the manuscript.

## Conflict of Interest Statement

The authors declare that the research was conducted in the absence of any commercial or financial relationships that could be construed as a potential conflict of interest.

## Footnotes

1 http://www.mirbase.org/ftp.shtml

2 http://rfam.sanger.ac.uk/search/

3 http://genie.weizmann.ac.il

4 http://www.genome.jp/kegg/

## Supplementary Material

FIGURE S1 A representative *Toxoplasma gondii* cyst detected in the homogenized mouse brain at 33 dpi.

FIGURE S2 List of the commonly expressed and specifically expressed miRNAs (*P* < 0.05) between the various mouse groups.

TABLE S1 Sequencing data of the known miRNAs during acute and chronic infection with *T. gondii* oocysts.

TABLE S2 Sequencing data of the novel miRNAs during acute and chronic infection with *T. gondii* oocysts.

TABLE S3 The total differentially expressed miRNAs (*P* < 0.05) during acute and chronic infection with *T. gondii* oocysts.

TABLE S4 List of the differentially expressed miRNAs in mouse brain following infection with oocysts and tissue cysts of *T. gondii* (PRU strain, type II).

## Abbreviations

AIDS: acquired immune deficiency syndrome
cAMP: cyclic adenosine monophosphate
cDNA: complementary deoxyribonucleic acid
CNS: central nervous system
CsCl: cesium chloride
DNA: deoxyribonucleic acid
dpi: days post-infection
FDR: false discovery rate
GO: gene ontology
IFN-α: interferon alpha
IFN-γ: interferon gamma
KEGG: Kyoto encyclopedia of genes and genomes
MAPK: mitogen-activated protein kinase
miRNA: micro-ribonucleic acid
PBS: phosphate buffered saline
PCR-RFLP: polymerase chain reaction-restriction fragment length polymorphism
qRT-PCR: quantitative reverse transcription polymerase chain reaction
RINs: ribonucleic acid integrity numbers
RNA: ribonucleic acid
RNA-seq: deep-sequencing analysis of small ribonucleic acids
rRNA: ribosomal ribonucleic acid
SD: standard deviation
snoRNA: small nucleolar ribonucleic acid
snRNA: small nuclear ribonucleic acid
SOCS1: suppressor of cytokine signaling 1
sRNA: small ribonucleic acid
TNF-α: tumor necrosis factor alpha
TPM: transcript per million
tRNA: transfer ribonucleic acid.
