# Supplementary figures and images for: Differential Brain MicroRNA Expression Profiles After Acute and Chronic Infection of Mice With Toxoplasma gondii Oocysts
Source: Front Microbiol. 2018 Oct 2;9:2316. doi: 10.3389/fmicb.2018.02316 (PMC6176049; doi:10.3389/fmicb.2018.02316)

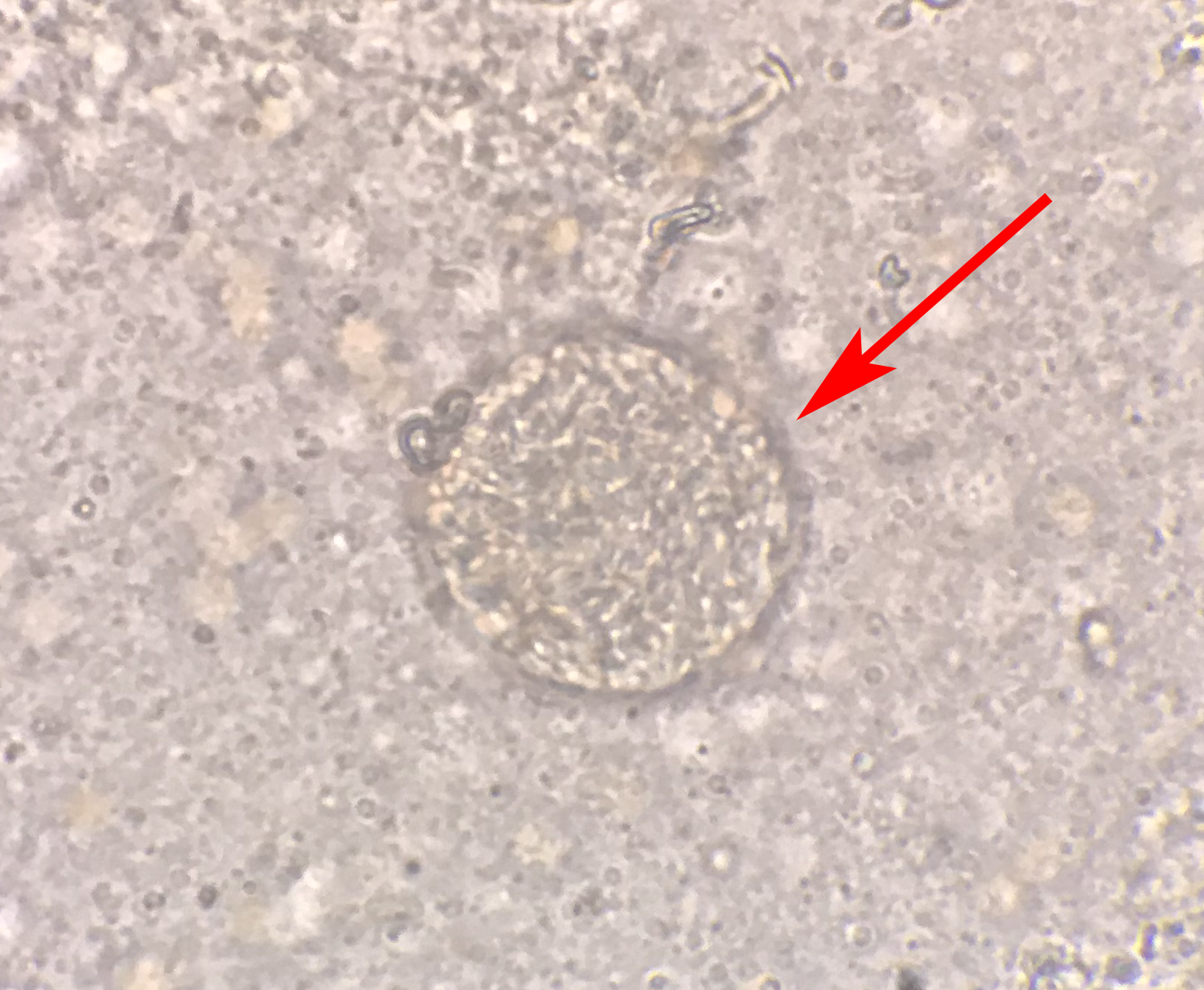

Supplement: FIGURE S1 — A representative Toxoplasma gondii cyst detected in the homogenized mouse brain at 33 dpi. [file Image_1.TIF]

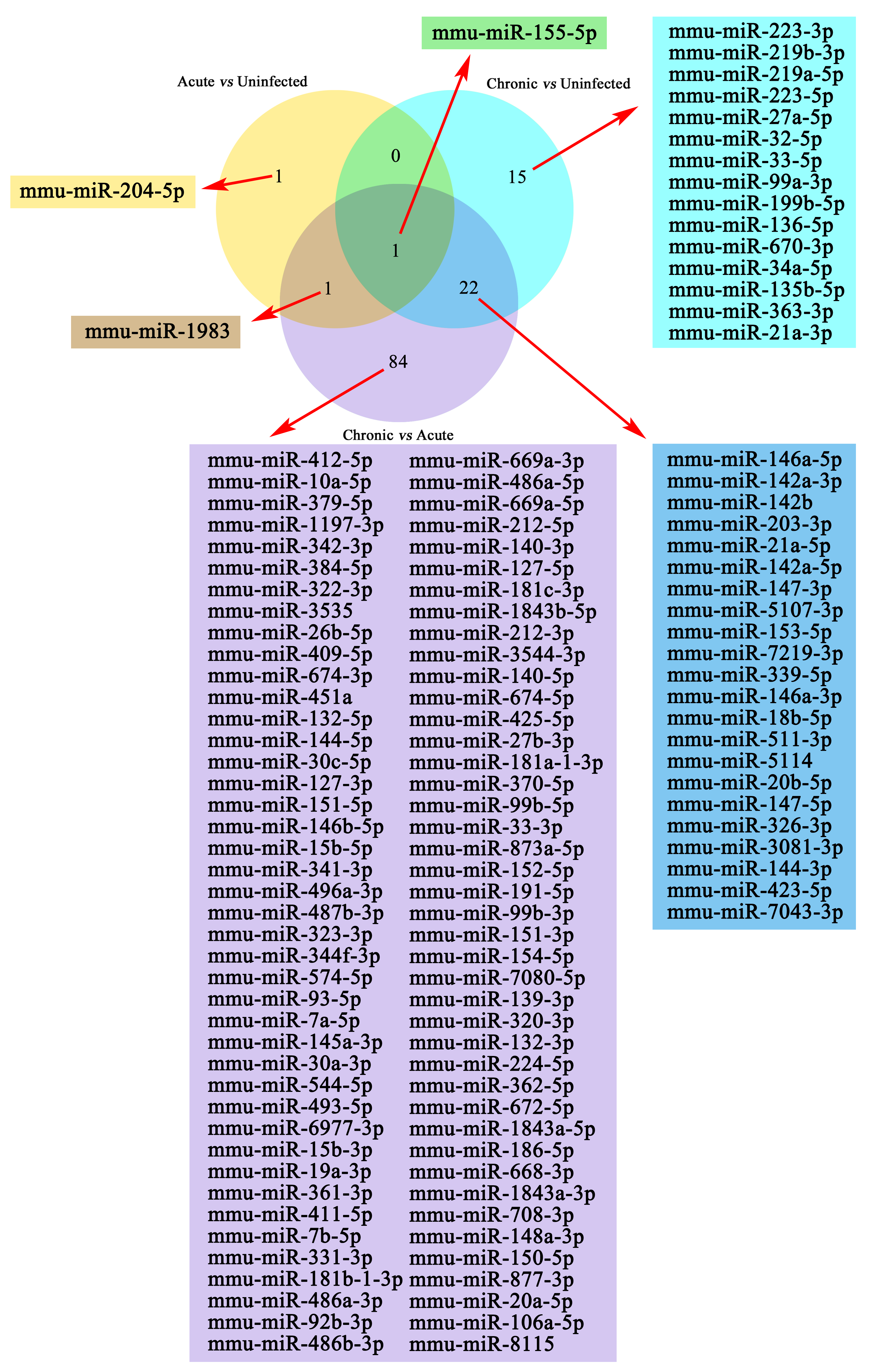

Supplement: FIGURE S2 — List of the commonly expressed and specifically expressed miRNAs (P < 0.05) between the various mouse groups. [file Image_2.TIF]
