# Supplementary material for: Differential Brain MicroRNA Expression Profiles After Acute and Chronic Infection of Mice With Toxoplasma gondii Oocysts
Source: Front Microbiol. 2018 Oct 2;9:2316. doi: 10.3389/fmicb.2018.02316 (PMC6176049; doi:10.3389/fmicb.2018.02316)
Supplement: TABLE S1 — Sequencing data of the known miRNAs during acute and chronic infection with T. gondii oocysts. [file Table_1.docx]

| **Table S1 \| Sequencing data of known miRNAs during acute and chronic infection with *T. gondii* oocysts.** | | | | | | | | | | | | | | | |
| --- | --- | --- | --- | --- | --- | --- | --- | --- | --- | --- | --- | --- | --- | --- | --- |
|  | 11 days post infection | | | | | | |  | 33 days post infection | | | | | | |
| Mouse groups | Control samples | | |  | Infected samples | | |  | Control samples | | |  | Infected samples | | |
| Mapped mature | 910 | 991 | 1,018 |  | 1,020 | 988 | 978 |  | 1,000 | 1,036 | 977 |  | 985 | 977 | 994 |
| Mapped hairpin | 681 | 732 | 748 |  | 762 | 744 | 730 |  | 739 | 763 | 727 |  | 746 | 742 | 748 |
| Mapped unique sRNA | 6,721 | 7,938 | 7,792 |  | 8,069 | 7,610 | 7,185 |  | 7,552 | 8,392 | 7,541 |  | 7,199 | 7,702 | 7,389 |
| Mapped total sRNA | 5,964,325 | 9,195,630 | 7,076,981 |  | 9,774,699 | 7,751,984 | 6,304,977 |  | 8,119,232 | 10,552,403 | 8,349,622 |  | 5,927,759 | 7,686,464 | 7,151,108 |
