# Supplementary material for: Differential Brain MicroRNA Expression Profiles After Acute and Chronic Infection of Mice With Toxoplasma gondii Oocysts
Source: Front Microbiol. 2018 Oct 2;9:2316. doi: 10.3389/fmicb.2018.02316 (PMC6176049; doi:10.3389/fmicb.2018.02316)
Supplement: TABLE S2 — Sequencing data of the novel miRNAs during acute and chronic infection with T. gondii oocysts. [file Table_2.docx]

| **Table S2 \| Sequencing data of novel miRNA mapping during acute and chronic infection with *T. gondii* oocysts.** | | | | | | | | | | | | | | | |
| --- | --- | --- | --- | --- | --- | --- | --- | --- | --- | --- | --- | --- | --- | --- | --- |
|  | 11 days post infection | | | | | | |  | 33 days post infection | | | | | | |
| Mouse groups | Control samples | | |  | Infected samples | | |  | Control samples | | |  | Infected samples | | |
| Mapped mature | 41 | 73 | 79 |  | 69 | 73 | 50 |  | 60 | 71 | 50 |  | 51 | 57 | 68 |
| Mapped star | 5 | 9 | 6 |  | 8 | 7 | 5 |  | 6 | 6 | 6 |  | 5 | 6 | 4 |
| Mapped hairpin | 48 | 77 | 84 |  | 73 | 78 | 54 |  | 62 | 73 | 54 |  | 54 | 61 | 69 |
| Mapped unique sRNA | 64 | 109 | 114 |  | 95 | 107 | 74 |  | 94 | 108 | 76 |  | 82 | 82 | 92 |
| Mapped total sRNA | 153 | 292 | 298 |  | 299 | 230 | 149 |  | 206 | 329 | 208 |  | 170 | 187 | 231 |
