# Supplementary material for: Differential Brain MicroRNA Expression Profiles After Acute and Chronic Infection of Mice With Toxoplasma gondii Oocysts
Source: Front Microbiol. 2018 Oct 2;9:2316. doi: 10.3389/fmicb.2018.02316 (PMC6176049; doi:10.3389/fmicb.2018.02316)
Supplement: TABLE S3 — The total differentially expressed miRNAs (P < 0.05) during acute and chronic infection with T. gondii oocysts. [file Table_3.docx]

**Table S3 | The total differentially expressed miRNAs (*P* < 0.05) during acute and chronic infection with *T. gondii* oocysts.**

| Mouse group | miRNA | Log _2_Fold change | *P*-value | *P-*adjustment | Regulation |
| --- | --- | --- | --- | --- | --- |
| acute *vs* uninfected |  |  |  |  |  |
|  | mmu-miR-155-5p | 1.3422 | 2.01E-06 | 0.001312 | Up-regulated |
|  | mmu-miR-1983 | 0.70165 | 2.55E-05 | 0.0083223 | Up-regulated |
|  | mmu-miR-204-5p | -0.73525 | 0.0001809 | 0.039436 | Down-regulated |
|  |  |  |  |  |  |
| chronic *vs* uninfected |  |  |  |  |  |
|  | mmu-miR-146a-5p | 3.7084 | 1.21E-61 | 9.77E-59 | Up-regulated |
|  | mmu-miR-155-5p | 4.0375 | 1.12E-43 | 4.54E-41 | Up-regulated |
|  | mmu-miR-142a-3p | 2.0541 | 1.00E-25 | 2.03E-23 | Up-regulated |
|  | mmu-miR-142b | 2.0541 | 1.00E-25 | 2.03E-23 | Up-regulated |
|  | mmu-miR-203-3p | 2.0275 | 1.77E-25 | 2.87E-23 | Up-regulated |
|  | mmu-miR-21a-5p | 1.9238 | 2.31E-23 | 3.11E-21 | Up-regulated |
|  | mmu-miR-142a-5p | 2.0311 | 4.23E-20 | 4.88E-18 | Up-regulated |
|  | mmu-miR-147-3p | 2.9077 | 3.22E-17 | 3.25E-15 | Up-regulated |
|  | mmu-miR-5107-3p | 2.9955 | 7.65E-13 | 6.86E-11 | Up-regulated |
|  | mmu-miR-223-3p | 1.838 | 8.64E-10 | 6.98E-08 | Up-regulated |
|  | mmu-miR-153-5p | 2.1254 | 7.40E-08 | 5.44E-06 | Up-regulated |
|  | mmu-miR-7219-3p | 2.1165 | 5.29E-07 | 3.56E-05 | Up-regulated |
|  | mmu-miR-339-5p | 0.66754 | 1.21E-05 | 0.00065036 | Up-regulated |
|  | mmu-miR-223-5p | 1.6953 | 1.75E-05 | 0.00088301 | Up-regulated |
|  | mmu-miR-27a-5p | 0.76656 | 5.92E-05 | 0.0026573 | Up-regulated |
|  | mmu-miR-146a-3p | 1.695 | 6.33E-05 | 0.0026901 | Up-regulated |
|  | mmu-miR-18b-5p | 1.6103 | 0.00016134 | 0.0062079 | Up-regulated |
|  | mmu-miR-511-3p | 1.349 | 0.00020702 | 0.0076033 | Up-regulated |
|  | mmu-miR-5114 | 1.5086 | 0.00043374 | 0.014018 | Up-regulated |
|  | mmu-miR-20b-5p | 1.0461 | 0.00047966 | 0.014906 | Up-regulated |
|  | mmu-miR-147-5p | 1.3139 | 0.00066673 | 0.019952 | Up-regulated |
|  | mmu-miR-423-5p | 0.44684 | 0.0010644 | 0.027398 | Up-regulated |
|  | mmu-miR-7043-3p | 1.0822 | 0.0010851 | 0.027398 | Up-regulated |
|  | mmu-miR-363-3p | 0.97175 | 0.0021014 | 0.046754 | Up-regulated |
|  | mmu-miR-21a-3p | 1.2277 | 0.0021994 | 0.046767 | Up-regulated |
|  | mmu-miR-219b-3p | -1.1693 | 1.63E-06 | 0.00010152 | Down-regulated |
|  | mmu-miR-219a-5p | -1.1559 | 1.97E-06 | 0.0001137 | Down-regulated |
|  | mmu-miR-32-5p | -0.43524 | 5.90E-05 | 0.0026573 | Down-regulated |
|  | mmu-miR-33-5p | -0.89719 | 9.15E-05 | 0.0036973 | Down-regulated |
|  | mmu-miR-99a-3p | -0.83122 | 0.00034778 | 0.012218 | Down-regulated |
|  | mmu-miR-199b-5p | -0.74691 | 0.00037218 | 0.01253 | Down-regulated |
|  | mmu-miR-326-3p | -0.42889 | 0.00071845 | 0.020732 | Down-regulated |
|  | mmu-miR-3081-3p | -0.97471 | 0.00078346 | 0.021829 | Down-regulated |
|  | mmu-miR-144-3p | -0.63175 | 0.0010824 | 0.027398 | Down-regulated |
|  | mmu-miR-136-5p | -0.50052 | 0.0015125 | 0.035944 | Down-regulated |
|  | mmu-miR-670-3p | -1.0838 | 0.0014777 | 0.035944 | Down-regulated |
|  | mmu-miR-34a-5p | -1.0817 | 0.0015596 | 0.036005 | Down-regulated |
|  | mmu-miR-135b-5p | -0.60951 | 0.0021409 | 0.046754 | Down-regulated |
| chronic *vs* acute |  |  |  |  |  |
|  | mmu-miR-146a-5p | 3.5491 | 3.24E-118 | 2.38E-115 | Up-regulated |
|  | mmu-miR-142a-3p | 2.6381 | 7.90E-56 | 1.93E-53 | Up-regulated |
|  | mmu-miR-142b | 2.6381 | 7.90E-56 | 1.93E-53 | Up-regulated |
|  | mmu-miR-155-5p | 3.0876 | 5.94E-28 | 7.26E-26 | Up-regulated |
|  | mmu-miR-142a-5p | 2.0908 | 1.26E-24 | 1.32E-22 | Up-regulated |
|  | mmu-miR-423-5p | 0.71476 | 2.92E-17 | 2.68E-15 | Up-regulated |
|  | mmu-miR-21a-5p | 1.6451 | 7.42E-15 | 6.04E-13 | Up-regulated |
|  | mmu-miR-147-3p | 2.5151 | 5.69E-14 | 4.17E-12 | Up-regulated |
|  | mmu-miR-10a-5p | 1.7159 | 7.83E-13 | 5.21E-11 | Up-regulated |
|  | mmu-miR-5107-3p | 2.7461 | 9.31E-10 | 4.87E-08 | Up-regulated |
|  | mmu-miR-342-3p | 0.67146 | 1.70E-09 | 8.28E-08 | Up-regulated |
|  | mmu-miR-384-5p | 0.47573 | 2.90E-09 | 1.33E-07 | Up-regulated |
|  | mmu-miR-203-3p | 1.2368 | 6.22E-08 | 2.68E-06 | Up-regulated |
|  | mmu-miR-3535 | 0.82288 | 2.11E-07 | 8.14E-06 | Up-regulated |
|  | mmu-miR-26b-5p | 0.41152 | 1.14E-06 | 4.18E-05 | Up-regulated |
|  | mmu-miR-451a | 1.3881 | 3.55E-06 | 0.00011324 | Up-regulated |
|  | mmu-miR-147-5p | 1.8433 | 4.32E-06 | 0.00013201 | Up-regulated |
|  | mmu-miR-144-5p | 1.397 | 5.74E-06 | 0.00016388 | Up-regulated |
|  | mmu-miR-30c-5p | 0.32312 | 6.35E-06 | 0.00017233 | Up-regulated |
|  | mmu-miR-153-5p | 1.7994 | 1.67E-05 | 0.00042084 | Up-regulated |
|  | mmu-miR-7043-3p | 1.403 | 1.95E-05 | 0.00044665 | Up-regulated |
|  | mmu-miR-146b-5p | 0.76158 | 2.72E-05 | 0.00059927 | Up-regulated |
|  | mmu-miR-15b-5p | 0.80873 | 2.78E-05 | 0.00059927 | Up-regulated |
|  | mmu-miR-7219-3p | 1.9398 | 3.43E-05 | 0.00071908 | Up-regulated |
|  | mmu-miR-574-5p | 0.84026 | 7.95E-05 | 0.0013882 | Up-regulated |
|  | mmu-miR-93-5p | 0.53577 | 7.78E-05 | 0.0013882 | Up-regulated |
|  | mmu-miR-7a-5p | 0.8422 | 9.44E-05 | 0.0016086 | Up-regulated |
|  | mmu-miR-145a-3p | 0.38496 | 0.0001164 | 0.0019391 | Up-regulated |
|  | mmu-miR-15b-3p | 1.253 | 0.00027933 | 0.0040368 | Up-regulated |
|  | mmu-miR-19a-3p | 1.2801 | 0.00027735 | 0.0040368 | Up-regulated |
|  | mmu-miR-361-3p | 0.27181 | 0.00028087 | 0.0040368 | Up-regulated |
|  | mmu-miR-7b-5p | 0.64998 | 0.00029526 | 0.004133 | Up-regulated |
|  | mmu-miR-146a-3p | 1.7927 | 0.00037389 | 0.004894 | Up-regulated |
|  | mmu-miR-339-5p | 0.51806 | 0.00047995 | 0.0060656 | Up-regulated |
|  | mmu-miR-486a-3p | 0.78717 | 0.0005302 | 0.0065871 | Up-regulated |
|  | mmu-miR-486b-3p | 0.78745 | 0.00061351 | 0.0072533 | Up-regulated |
|  | mmu-miR-669a-3p | 0.44317 | 0.0006128 | 0.0072533 | Up-regulated |
|  | mmu-miR-486a-5p | 0.80364 | 0.00063959 | 0.0074416 | Up-regulated |
|  | mmu-miR-669a-5p | 0.78728 | 0.00065842 | 0.0075409 | Up-regulated |
|  | mmu-miR-140-3p | 0.36312 | 0.00073554 | 0.008169 | Up-regulated |
|  | mmu-miR-140-5p | 0.52449 | 0.0014416 | 0.014475 | Up-regulated |
|  | mmu-miR-425-5p | 0.46328 | 0.0015136 | 0.014992 | Up-regulated |
|  | mmu-miR-20b-5p | 1.1881 | 0.0021036 | 0.019518 | Up-regulated |
|  | mmu-miR-144-3p | 0.9185 | 0.0026007 | 0.023402 | Up-regulated |
|  | mmu-miR-152-5p | 0.72345 | 0.0026698 | 0.023578 | Up-regulated |
|  | mmu-miR-191-5p | 0.41995 | 0.0027459 | 0.02394 | Up-regulated |
|  | mmu-miR-7080-5p | 1.294 | 0.0034081 | 0.028388 | Up-regulated |
|  | mmu-miR-320-3p | 0.48949 | 0.0036497 | 0.029725 | Up-regulated |
|  | mmu-miR-511-3p | 1.0402 | 0.0040597 | 0.032701 | Up-regulated |
|  | mmu-miR-5114 | 1.4209 | 0.0041202 | 0.032827 | Up-regulated |
|  | mmu-miR-224-5p | 1.2727 | 0.0043463 | 0.033185 | Up-regulated |
|  | mmu-miR-362-5p | 0.41317 | 0.0043303 | 0.033185 | Up-regulated |
|  | mmu-miR-672-5p | 0.45289 | 0.0042901 | 0.033185 | Up-regulated |
|  | mmu-miR-186-5p | 0.18237 | 0.0052345 | 0.039152 | Up-regulated |
|  | mmu-miR-148a-3p | 0.50228 | 0.0058557 | 0.042081 | Up-regulated |
|  | mmu-miR-20a-5p | 0.50687 | 0.0063673 | 0.04445 | Up-regulated |
|  | mmu-miR-18b-5p | 1.3395 | 0.0067634 | 0.046769 | Up-regulated |
|  | mmu-miR-106a-5p | 1.1189 | 0.0070924 | 0.048586 | Up-regulated |
|  | mmu-miR-8115 | 1.1062 | 0.0072883 | 0.049466 | Up-regulated |
|  | mmu-miR-412-5p | -1.4876 | 8.50E-46 | 1.56E-43 | Down-regulated |
|  | mmu-miR-1983 | -1.1776 | 5.92E-28 | 7.26E-26 | Down-regulated |
|  | mmu-miR-379-5p | -0.61196 | 6.21E-11 | 3.79E-09 | Down-regulated |
|  | mmu-miR-1197-3p | -1.3826 | 7.61E-11 | 4.29E-09 | Down-regulated |
|  | mmu-miR-322-3p | -0.71614 | 1.41E-07 | 5.74E-06 | Down-regulated |
|  | mmu-miR-409-5p | -0.63403 | 1.95E-06 | 6.49E-05 | Down-regulated |
|  | mmu-miR-674-3p | -0.6952 | 1.87E-06 | 6.49E-05 | Down-regulated |
|  | mmu-miR-132-5p | -0.58255 | 5.81E-06 | 0.00016388 | Down-regulated |
|  | mmu-miR-127-3p | -0.31406 | 1.48E-05 | 0.00038677 | Down-regulated |
|  | mmu-miR-3081-3p | -0.90774 | 1.73E-05 | 0.00042203 | Down-regulated |
|  | mmu-miR-151-5p | -0.7486 | 1.89E-05 | 0.00044665 | Down-regulated |
|  | mmu-miR-341-3p | -0.67422 | 5.29E-05 | 0.0010481 | Down-regulated |
|  | mmu-miR-496a-3p | -0.48463 | 5.22E-05 | 0.0010481 | Down-regulated |
|  | mmu-miR-487b-3p | -0.52368 | 6.47E-05 | 0.0012486 | Down-regulated |
|  | mmu-miR-323-3p | -0.52399 | 7.80E-05 | 0.0013882 | Down-regulated |
|  | mmu-miR-344f-3p | -0.66609 | 7.65E-05 | 0.0013882 | Down-regulated |
|  | mmu-miR-30a-3p | -0.38317 | 0.00016205 | 0.0026365 | Down-regulated |
|  | mmu-miR-544-5p | -0.69322 | 0.00016545 | 0.0026365 | Down-regulated |
|  | mmu-miR-493-5p | -0.54134 | 0.00017171 | 0.0026779 | Down-regulated |
|  | mmu-miR-6977-3p | -1.2936 | 0.00022206 | 0.003391 | Down-regulated |
|  | mmu-miR-411-5p | -0.38288 | 0.00029884 | 0.004133 | Down-regulated |
|  | mmu-miR-331-3p | -0.60923 | 0.00031559 | 0.0042838 | Down-regulated |
|  | mmu-miR-181b-1-3p | -0.65654 | 0.00034504 | 0.0045984 | Down-regulated |
|  | mmu-miR-326-3p | -0.45061 | 0.0004591 | 0.0059038 | Down-regulated |
|  | mmu-miR-92b-3p | -0.29309 | 0.00054955 | 0.0067136 | Down-regulated |
|  | mmu-miR-212-5p | -0.37937 | 0.00067754 | 0.0076406 | Down-regulated |
|  | mmu-miR-127-5p | -0.43157 | 0.00099006 | 0.010747 | Down-regulated |
|  | mmu-miR-181c-3p | -0.53492 | 0.00099699 | 0.010747 | Down-regulated |
|  | mmu-miR-1843b-5p | -0.24246 | 0.0010124 | 0.010755 | Down-regulated |
|  | mmu-miR-212-3p | -0.76224 | 0.001386 | 0.014309 | Down-regulated |
|  | mmu-miR-3544-3p | -0.8833 | 0.0013679 | 0.014309 | Down-regulated |
|  | mmu-miR-674-5p | -0.527 | 0.0014414 | 0.014475 | Down-regulated |
|  | mmu-miR-27b-3p | -0.37739 | 0.0015629 | 0.015274 | Down-regulated |
|  | mmu-miR-181a-1-3p | -0.36347 | 0.0016594 | 0.016004 | Down-regulated |
|  | mmu-miR-370-5p | -0.49908 | 0.0020663 | 0.019518 | Down-regulated |
|  | mmu-miR-99b-5p | -0.26011 | 0.0020835 | 0.019518 | Down-regulated |
|  | mmu-miR-33-3p | -0.66244 | 0.002605 | 0.023402 | Down-regulated |
|  | mmu-miR-873a-5p | -0.56375 | 0.0026179 | 0.023402 | Down-regulated |
|  | mmu-miR-99b-3p | -0.29922 | 0.0027761 | 0.02394 | Down-regulated |
|  | mmu-miR-151-3p | -0.29263 | 0.0030598 | 0.02608 | Down-regulated |
|  | mmu-miR-154-5p | -0.45786 | 0.0032185 | 0.027117 | Down-regulated |
|  | mmu-miR-139-3p | -0.36701 | 0.003513 | 0.028933 | Down-regulated |
|  | mmu-miR-132-3p | -0.59646 | 0.0042071 | 0.033159 | Down-regulated |
|  | mmu-miR-1843a-5p | -0.27617 | 0.0044674 | 0.033759 | Down-regulated |
|  | mmu-miR-668-3p | -0.3682 | 0.0052974 | 0.039222 | Down-regulated |
|  | mmu-miR-1843a-3p | -0.46488 | 0.0053906 | 0.039255 | Down-regulated |
|  | mmu-miR-708-3p | -0.34334 | 0.0054089 | 0.039255 | Down-regulated |
|  | mmu-miR-150-5p | -0.41899 | 0.0060423 | 0.043 | Down-regulated |
|  | mmu-miR-877-3p | -0.52123 | 0.0061426 | 0.043293 | Down-regulated |
