# Supplementary material for: Differential Brain MicroRNA Expression Profiles After Acute and Chronic Infection of Mice With Toxoplasma gondii Oocysts
Source: Front Microbiol. 2018 Oct 2;9:2316. doi: 10.3389/fmicb.2018.02316 (PMC6176049; doi:10.3389/fmicb.2018.02316)
Supplement: TABLE S4 — List of the differentially expressed miRNAs in mouse brain following infection with oocysts and tissue cysts of T. gondii (PRU strain, type II). [file Table_4.docx]

**TABLE S4 |** **List of the differentially expressed miRNAs (*P* < 0.05) in mouse brain following infection with oocysts and tissue cysts of *T. gondii* (PRU strain, type II).**

| *T. gondii* oocysts (PRU strain)  The present study | |  | *T. gondii* tissue cysts (PRU strain)  Xu et al., (2013) | |
| --- | --- | --- | --- | --- |
| 11 dpi | 33 dpi |  | 14 dpi | 21 dpi |
| mmu-miR-155-5p | mmu-miR-146a-5p |  | mmu-miR-9 | mmu-miR-694 |
| mmu-miR-1983 | mmu-miR-155-5p |  | mmu-miR-470 | mmu-miR-5101 |
| mmu-miR-204-5p | mmu-miR-142a-3p |  | mmu-miR-3105-5p | mmu-miR-1955-5p |
|  | mmu-miR-142b |  | mmu-miR-290-5p | mmu-miR-1196 |
|  | mmu-miR-203-3p |  | mmu-miR-546 | mmu-miR-18a |
|  | mmu-miR-21a-5p |  | mmu-miR-1936 | mmu-miR-599 |
|  | mmu-miR-142a-5p |  | mmu-miR-5103 | mmu-miR-741 |
|  | mmu-miR-147-3p |  | mmu-miR-1932 | mmu-miR-875-5p |
|  | mmu-miR-5107-3p |  | mmu-miR-5126 | mmu-miR-3544 |
|  | mmu-miR-223-3p |  | mmu-miR-nov-14d-speci-1 | mmu-miR-3091-5p |
|  | mmu-miR-153-5p |  | mmu-miR-nov-14d-speci-2 | mmu-miR-18b |
|  | mmu-miR-7219-3p |  | mmu-miR-nov-14d-speci-3 | mmu-miR-466f |
|  | mmu-miR-219b-3p |  | mmu-miR-nov-14d-speci-4 | mmu-miR-719 |
|  | mmu-miR-219a-5p |  | mmu-miR-nov-14d-speci-5 | mmu-miR-nov-21d-speci-1 |
|  | mmu-miR-339-5p |  | mmu-miR-nov-14d-speci-6 | mmu-miR-nov-21d-speci-2 |
|  | mmu-miR-223-5p |  | mmu-miR-nov-14d-speci-7 | mmu-miR-nov-21d-speci-3 |
|  | mmu-miR-27a-5p |  | mmu-miR-nov-14d-speci-8 | mmu-miR-nov-21d-speci-4 |
|  | mmu-miR-32-5p |  |  | mmu-miR-nov-21d-speci-6 |
|  | mmu-miR-146a-3p |  |  | mmu-miR-nov-21d-speci-8 |
|  | mmu-miR-33-5p |  |  | mmu-miR-nov-21d-speci-9 |
|  | mmu-miR-18b-5p |  |  | mmu-miR-nov-21d-speci-10 |
|  | mmu-miR-511-3p |  |  | mmu-miR-nov-21d-speci-11 |
|  | mmu-miR-99a-3p |  |  | mmu-miR-nov-21d-speci-12 |
|  | mmu-miR-199b-5p |  |  | mmu-miR-nov-21d-speci-13 |
|  | mmu-miR-5114 |  |  | mmu-miR-nov-21d-speci-14 |
|  | mmu-miR-20b-5p |  |  | mmu-miR-nov-21d-speci-15 |
|  | mmu-miR-147-5p |  |  | mmu-miR-nov-21d-speci-16 |
|  | mmu-miR-326-3p |  |  | mmu-miR-nov-21d-speci-17 |
|  | mmu-miR-3081-3p |  |  | mmu-miR-nov-21d-speci-18 |
|  | mmu-miR-144-3p |  |  | mmu-miR-nov-21d-speci-19 |
|  | mmu-miR-423-5p |  |  | mmu-miR-nov-21d-speci-20 |
|  | mmu-miR-7043-3p |  |  |  |
|  | mmu-miR-136-5p |  |  |  |
|  | mmu-miR-670-3p |  |  |  |
|  | mmu-miR-34a-5p |  |  |  |
|  | mmu-miR-135b-5p |  |  |  |
|  | mmu-miR-363-3p |  |  |  |
|  | mmu-miR-21a-3p |  |  |  |
